# The Attenuation of Traumatic Brain Injury via Inhibition of Oxidative Stress and Apoptosis by Tanshinone IIA

**DOI:** 10.1155/2020/4170156

**Published:** 2020-05-01

**Authors:** Yongpan Huang, Xian Long, Jiayu Tang, Xinliang Li, Xiang Zhang, Chunyan Luo, Yan Zhou, Pan Zhang

**Affiliations:** ^1^Department of Clinic, Medicine School, Changsha Social Work College, Changsha, Hunan, China; ^2^Department of Pharmacology, Institute of Chinese Medicine, Hunan Academy of Chinese Medicine, Changsha, China; ^3^Department of Neurology, Brain Hospital of Hunan Province, Changsha, China

## Abstract

Traumatic brain injury (TBI) is a major source of mortality and long-term disability worldwide. The mechanisms associated with TBI development are poorly understood, and little progress has been made in the treatment of TBI. Tanshinone IIA is an effective agent to treat a variety of disorders; however, the mechanisms of Tanshinone IIA on TBI remain unclear. The aim of the present study was to investigate the therapeutic potential of Tanshinone IIA on TBI and its underlying molecular mechanisms. Changes in microvascular permeability were examined to determine the extent of TBI with Evans blue dye. Brain edema was assessed by measuring the wet weight to dry weight ratio. The expression levels of CD11, interleukin- (IL-) 1*β*, and tumor necrosis factor- (TNF-) *α* mRNA were determined by reverse transcription-quantitative PCR. Aquaporin-4 (AQP4), glial fibrillary acidic protein (GFAP), and p47phox protein expression levels were detected by western blotting. Superoxide dismutase (SOD), catalase and glutathione peroxidase (GSH-PX) activities, and malondialdehyde (MDA) content were determined using commercial kits. Cell apoptosis was detected by western blotting and TUNEL staining. Tanshinone IIA (10 mg/kg/day, intraperitoneal administration) significantly reduced brain water content and vascular permeability at 12, 24, 48, and 72 h after TBI. Tanshinone IIA downregulated the mRNA expression levels of various factors induced by TBI, including CD11, IL-1*β*, and TNF-*α*. Notably, CD11 mRNA downregulation suggested that Tanshinone IIA inhibited microglia activation. Further results showed that Tanshinone IIA treatment significantly downregulated AQP4 and GFAP expression. TBI-induced oxidative stress and apoptosis were markedly reversed by Tanshinone IIA, with an increase in SOD and GSH-PX activities and a decrease in the MDA content. Moreover, Tanshinone IIA decreased TBI-induced NADPH oxidase activation via the inhibition of p47phox. Tanshinone IIA attenuated TBI, and its mechanism of action may involve the inhibition of oxidative stress and apoptosis.

## 1. Introduction

Traumatic brain injury (TBI) is the most common cause of mortality and disability worldwide, and the damages induced by TBI can worsen the quality of life of the patients [1]. The brain damage following TBI is characterized by two phases, the initial, or primary, phase is characterized by direct cerebral tissue damage which results in glutamate release, calcium homeostasis disruption, N-methyl-D-aspartate receptor activation, permeability increase, and consecutive edema formation, which is an important self-protective mechanism to minimize the extent of the damage immediately after TBI. Importantly, the first response phase involves diverse cellular and molecular mechanisms that are important to maintain the homeostasis of the damaged tissue [2]. These events may cause cellular structural damage, neuronal cell death, oxidative stress, brain edema, blood-brain barrier (BBB) breakdown, and inflammation [3]. Among these adverse factors, edema formation is considered to be a key to the consequences of TBI, which may deteriorate the prognosis. Accumulating evidence from clinical and experimental studies has expanded the current knowledge of the pathophysiological phenomena underlying TBI and may facilitate the development of novel treatments with neuroprotective effects [4, 5].

**Figure 1:**
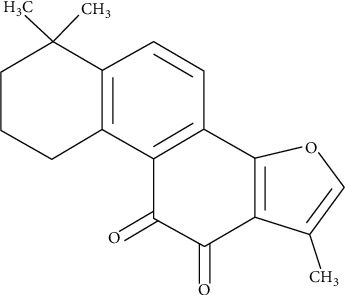
Structure of Tanshinone IIA.

Tanshinone IIA (Figure 1), a derivative of phenanthrenequinone derived from *Salvia miltiorrhiza* BUNGE (Danshen), possesses various pharmacological properties. A previous study showed that Tanshinone IIA is widely used in the treatment of cardiovascular and cerebrovascular diseases, inflammation, cholinesterase, collagenase, platelet aggregation, and cancer, due to its antioxidative activity [5–10]. Several previous studies showed that Tanshinone IIA has a protective effect by scavenging lipid free radicals, thus decreasing cytotoxicity *in vitro* and *in vivo* [11–14]. Further studies confirmed that Tanshinone IIA has significant protective effects against A*β*-induced neurotoxicity in cultured cortical neurons and PC12 cells [15, 16]. A recent study showed that Tanshinone IIA could improve memory deficits by acting on the hippocampus in STZ-induced diabetic mice [17]. However, whether Tanshinone IIA could mitigate TBI remains unknown. Therefore, the present study is aimed at investigating the protective roles and the mechanisms of Tanshinone IIA in a rat model of TBI.

## 2. Materials and Methods

### 2.1. Animal Studies and Ethics Statement

Male Sprague-Dawley rats (weight, 220-250 g) were used in the present experiments. Animals had free access to food and water and were maintained in plastic cages at 21 ± 2°C under a 12 h light/dark cycle. All the animal experiments were approved by Hunan Academy of Chinese Medicine Animal Care and Use Committee (approval no. Xiang 2019-0013). The animals were randomly assigned to the following groups: (i) Sham group (*n* = 24), (ii) TBI group (*n* = 24), and (iii) TBI+Tanshinone IIA (10 mg/kg/day) group (*n* = 24). After TBI, rats were immediately treated with PBS or 10 mg/kg Tanshinone IIA intraperitoneally as previously described [18]. At 12, 24, 48, and 72 h following trauma, all animals were sacrificed for further analysis. In total, six animals were analyzed in each group.

### 2.2. TBI Model Establishment

A traumatic brain injury model was established as previously described [18]. The rats were anesthetized and fixed in prone position, and the skull was cut sagittally. The injury was performed using a weight to damage the exposed area, 1.5 mm behind the coronal suture. A 2.5 mm diameter channel was drilled 2.5 mm to the right of the sagittal suture. The injury was performed in order to damage the bone window keeping the dura mater intact. The height of the falling weight was modulated to induce different degrees of contusion in the right parietal lobe. After TBI, all animals were kept and monitored until spontaneous respiration was reestablished. Sham group animals underwent anesthesia and scalp incision, but without TBI.

### 2.3. Determination of Antioxidant Indices in Tissues

The tissue levels of antioxidant indices including SOD, GSH-PX, CAT, and MDA were measured by commercial kits according to the instruction of manufacturer.

### 2.4. Determination of Brain Edema

The wet weight (WW) to dry weight (DW) ratio method was used to evaluate brain water content, as previously described [19]. The right brain hemispheres were rapidly removed from all animals postmortem at the indicated time points after TBI. Brains were weighed (to measure the WW), dried at 70°C for 72 h, and weighed again (to measure the DW). The percentage of tissue water content was calculated as (WW − DW)/WW × 100%.

### 2.5. BBB Breakdown Evaluation

Alterations in the microvascular permeability were examined to determine the extent of TBI by Evans blue dye (0.2 ml/100 g), which was injected through the femoral vein. Following anesthesia with 1% pentobarbital sodium (30-40 mg/kg), the thoracic cavity was exposed, intracardiac perfusion was performed with heparin saline, and the brain tissue was weighed, cut, and placed in dimethylformamide for 60 h at 60°C, centrifuged at 1,000 rpm for 5 min, and the absorbance at a wavelength of 620 nm was measured with a spectrophotometer. Data analysis was performed using Origin software (version s7.0), and the Evans blue content was calculated from the standard curve previously plotted.

### 2.6. TUNEL Staining

The formalin-fixed frontal cortex tissues were embedded in paraffin and sectioned (thickness, 4 *μ*m). In total, five brain regions from each group were cut with a microtome. The sections were analyzed by TUNEL assay to detect the apoptotic rate. The TUNEL assay kit was purchased from Roche Molecular Biochemicals, and the experiment was conducted according to the manufacturer's protocol. Apoptotic cells with condensed nuclei were stained green, while normal cells were large, round, and not stained. The positive cells were analyzed under a fluorescence microscope by a blinded investigator. The extent of brain injury was evaluated by measuring the rate of TUNEL-positive cells.

### 2.7. Real-Time PCR

The gene expression level in brain tissues was determined by real-time PCR. Extracted total RNA was purified with 75% ethanol, and its concentration was determined by spectrophotometry. Then, the purified total RNA (200 ng) was retrotranscribed using a retrotranscription kit (DRR037A; Takara Bio, Inc.) and mixed to obtain the first-strand cDNA template. Subsequently, the expression levels of CD68, interleukin- (IL-)1*β*, and tumor necrosis factor- (TNF-) *α* were determined quantitatively by real-time PCR (ABI 7300) using the SYBR Premix Ex Taq kit (Takara Bio, Inc.).

### 2.8. Western Blotting

For western blotting analysis, 40 *μ*g protein (from each sample) was separated by SDS-PAGE (10% gel). The gel was transferred to a PVDF membrane, which was blocked with 5% milk powder and incubated with rabbit anti-aquaporin-4 (AQP4), glial fibrillary acidic protein (GFAP), and anti-p47phox at 4°C overnight. Subsequently, the membranes were washed and incubated with horseradish peroxidase-conjugated secondary antibodies, and the protein bands were developed using an ECL kit (Amersham Biosciences). The expression levels of the proteins were calculated using a Molecular Imager ChemiDoc XRS System (Bio-Rad Laboratories, Inc.). The protein expression level was normalized to *β*-actin.

### 2.9. Statistical Analysis

For statistical analysis, the SPSS software (version 21.0) was used. Data are presented as the mean ± SEM. The data were analyzed by one-way ANOVA. *P* < 0.05 was considered to indicate a statistically significant difference.

## 3. Results

### 3.1. Effect of Tanshinone IIA on Brain Tissue Water Content and Vascular Permeability

**Figure 2:**
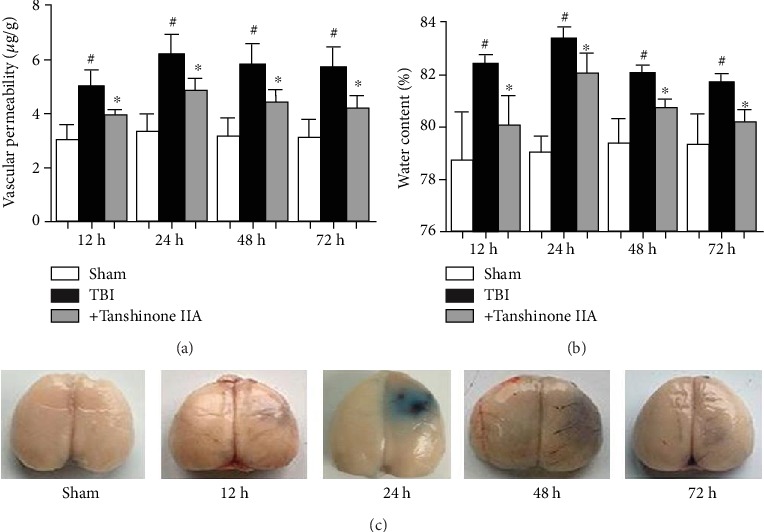
Tanshinone IIA attenuates brain tissue water content and vascular permeability. (a) Evans blue dye (0.2 ml/100 g) was injected through the femoral vein. (b) Brain tissue water content was analyzed and the percentage of tissue water content was calculated with the following formula: (WW − DW)/WW × 100%. (c) Evans blue staining of brain samples at 0, 12, 24, 48, and 72 h after TBI. Data are expressed as the mean ± SEM. *n* = 6 in each group. ^#^*P* < 0.05 vs. Sham group; ^∗^*P* < 0.05 vs. Tanshinone IIA group. TBI: traumatic brain injury; WW: wet weight; DW: dry weight.

To test whether Tanshinone IIA treatment exhibited neuroprotective effects after TBI, rats were sacrificed after treatment with or without Tanshinone IIA. As shown in Figure 2(c), the brain tissue water content increased significantly at 12, 24, 48, and 72 h in the TBI group, in particular, at 24 h.

The BBB is a specialized structure in the central nervous system that can block the entry of macromolecular substances from the peripheral blood into the brain parenchyma, thus maintaining cerebral homeostasis. The TBI-derived brain damage may alter the BBB permeability, promoting inflammation. Therefore, by measuring the amount of Evans blue dye in the brain, it is possible to assess the BBB breakdown and the vascular permeability in the brain tissue. The vascular permeability after TBI was examined to investigate whether Tanshinone IIA exerted its protective effect on the integrity of BBB structure and function following TBI. As shown in Figures 2(b) and 2(c), compared with the Sham group, the vascular permeability was significantly increased in the TBI group, which was reversed by Tanshinone IIA treatment.

### 3.2. Effect of Tanshinone IIA on the Expression Levels of AQP4 and GFAP

**Figure 3:**
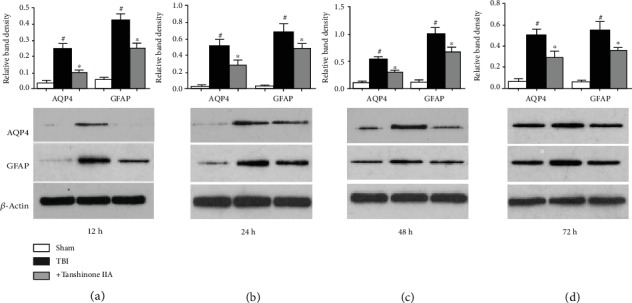
Effects of Tanshinone IIA on the expression levels of AQP4 and GFAP following TBI. (a) Western blotting analysis of AQP4 and GFAP at 12 h. (b) Western blotting analysis of AQP4 and GFAP at 24 h. (c) Western blotting analysis of AQP4 and GFAP at 48 h. (d) Western blotting analysis of AQP4 and GFAP at 72 h. Data are expressed as the mean ± SEM. *n* = 6 in each group. ^#^*P* < 0.05 vs. Sham group; ^∗^*P* < 0.05 vs. Tanshinone IIA group. TBI: traumatic brain injury; AQP4: aquaporin 4; GFAP: glial fibrillary acidic protein.

As the main water transporter in the central nervous system, AQP4 is involved in maintaining the water homeostasis in the BBB, and it is responsible for the formation of the vasogenic edema following TBI. To determine the effect of BBB impairment following TBI, the expression of AQP4 was investigated using western blotting analysis. As shown in Figure 3, TBI caused significant increases in AQP4 expression after 12, 24, 48, and 72 h. Tanshinone IIA treatment significantly decreased the expression level of AQP4, suggesting that Tanshinone IIA could regulate the permeability of BBB by regulating the expression levels of the proteins associated with this process. The present findings suggested that Tanshinone IIA could promote the repair of the BBB.

GFAP is only expressed in the perinuclear region and cytoplasm of mature glial cells in the central nervous system (CNS); it is involved in the formation of astrocyte cytoskeleton and has been used as surrogate marker of intracranial sequelae after TBI [20]. Therefore, the expression levels of GFAP were assessed by western blotting. As shown in Figure 3, TBI caused an increase in GFAP expression. Tanshinone IIA significantly reversed TBI-induced GFAP upregulation. The present results suggested that Tanshinone IIA alleviated TBI-induced injury.

### 3.3. Effect of Tanshinone IIA on TBI-Induced Microglia Activation

**Figure 4:**
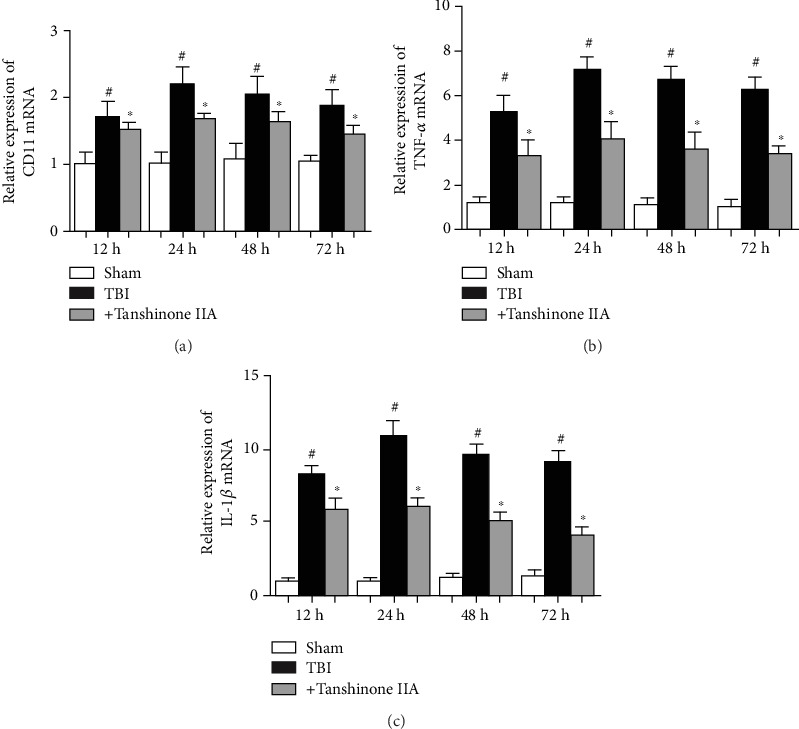
Effects of Tanshinone IIA on TBI-induced microglia activation. (a) RT-PCR analysis of the levels of CD11 expression. (b) RT-PCR analysis of the levels of TNF-*α* expression. (c) RT-PCR analysis of the levels of IL-1*β* expression. Data are expressed as the mean ± SEM. *n* = 6 in each group. ^#^*P* < 0.05 vs. Sham group; ^∗^*P* < 0.05 vs. Tanshinone IIA group. TBI: traumatic brain injury; RT: real-time.

Microglia activation occurs after TBI and is considered to be an important factor underlying the damage induced by TBI. Moreover, microglia activation is associated with upregulation of IL-1*β* and TNF-*α*, thus aggravating TBI and increasing the risk of stroke. To assess microglia activation, western blotting was performed on the brain tissues at 24 h after TBI, and the expression level of the microglia activation marker CD11 was examined. As shown in Figure 4, the expression of CD11 mRNA, a marker of microglia, was increased in the brain tissue after TBI. Moreover, TNF-*α* and IL-1*β* mRNA expressions increased. However, the treatment with Tanshinone IIA downregulated the mRNA expression levels of CD11, TNF-*α*, and IL-1*β*, suggesting that Tanshinone IIA inhibited TBI-induced microglia activation.

### 3.4. Effect of Tanshinone IIA on TBI-Induced Caspase-3 Activation

**Figure 5:**
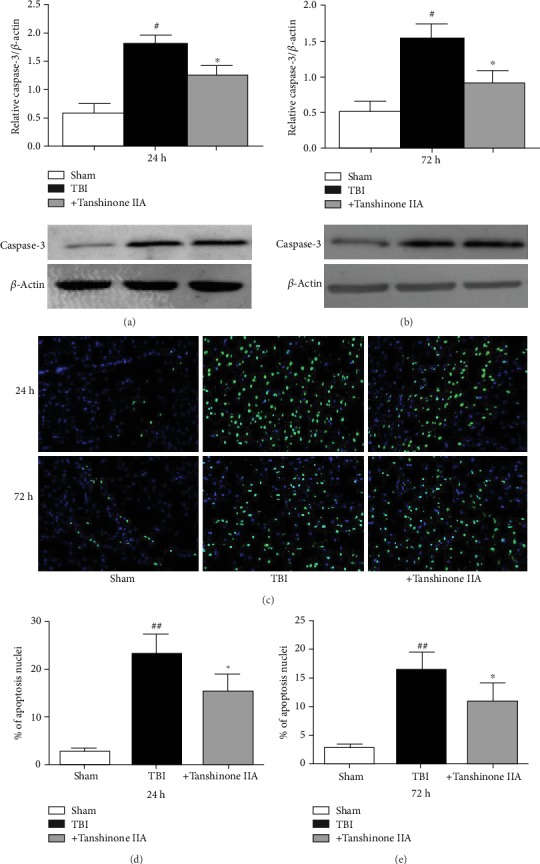
Tanshinone IIA alleviates TBI-induced apoptosis. (a, b) Western blotting analysis of Caspase-3 at 24 and 72 h. (c) TUNEL staining. Magnification, ×200. (d, e) Apoptotic rate. Data are expressed as the mean ± SEM. *n* = 6 in each group. ^#^*P* < 0.05 vs. Sham group; ^∗^*P* < 0.05 vs. Tanshinone IIA group. TBI: traumatic brain injury.

Accumulating evidence demonstrated that the caspase cascade is involved in a variety of biological processes, including the initiation of apoptosis. Caspase-3 expression was analyzed to investigate whether caspase-3 was involved in TBI-induced apoptosis in the brain. As shown in Figures 5(a) and 5(b), TBI caused a significant increase in caspase-3 activation. By contrast, the caspase-3 level, upregulated by TBI, was downregulated by Tanshinone IIA treatment.

To further assess the effects of Tanshinone IIA on apoptosis, TUNEL staining was performed. As shown in Figures 5(c)–5(e), TBI increased cell apoptosis, as assessed by morphological alterations, including nuclear fragmentation and apoptotic bodies, typical of cells undergoing apoptosis. Tanshinone IIA decreased the number of apoptotic cells. The present results suggested that Tanshinone IIA exhibited protective effects on TBI by inhibiting the apoptotic pathway.

### 3.5. Effect of Tanshinone IIA on TBI-Induced Oxidative Stress

**Figure 6:**
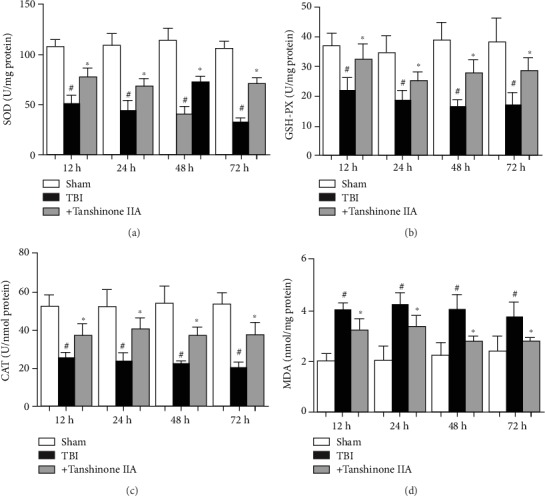
Effects of Tanshinone IIA on antioxidant levels in brain tissues. Measurement of (a) SOD, (b) GSH-PX, and (c) CAT activity. (d) MDA content. Data are expressed as the mean ± SEM. *n* = 6 in each group. ^#^*P* < 0.05 vs. Sham group; ^∗^*P* < 0.05 vs. Tanshinone IIA group. TBI: traumatic brain injury; SOD: superoxide dismutase; CAT: catalase; GSH-PX: glutathione peroxidase; MDA: malondialdehyde.

To determine the protective effect of Tanshinone IIA on TBI rats, various antioxidant factors, including superoxide dismutase (SOD), catalase (CAT), and glutathione peroxidase (GSH-PX), were examined. As shown in Figures 6(a)–6(d), compared with the Sham group, TBI caused decreases in the expression levels of SOD, CAT, and GSH-PX and an increase in MDA content. Tanshinone IIA significantly increased the activities of SOD, CAT, and GSH-PX and decreased MDA content. The present results suggested that Tanshinone IIA reduced oxidative stress.

**Figure 7:**
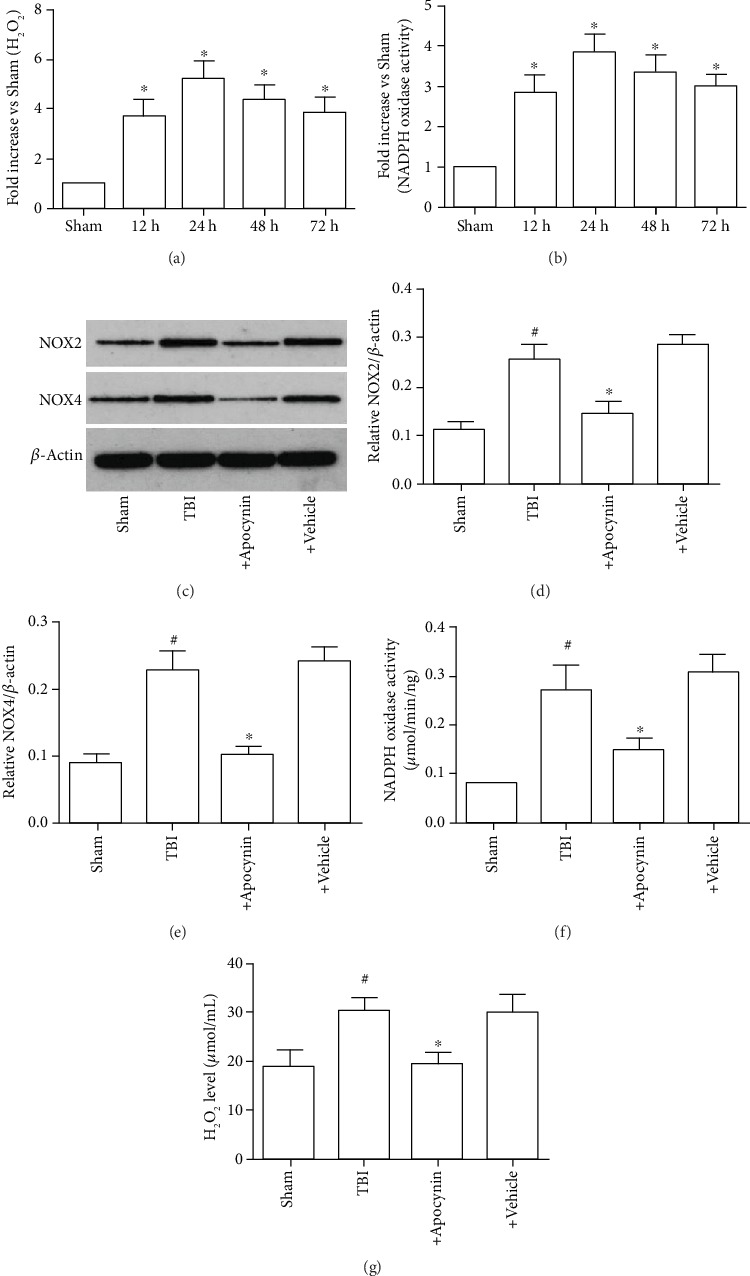
Induction of NADPH oxidase activation and O_2_^−^ generation in the brain following TBI. (a) NADPH oxidase activity at 0, 12, 24, 48, and 72 h after TBI. (b) O_2_^−^ production in the cerebral cortex at 0, 12, 24, 48, and 72 h after TBI. (c) Expression levels of NOX2 and NOX4. (d) Expression levels of NOX2. (e) Expression levels of NOX4. (f) NADPH oxidase activity at 24 h after TBI. (g) O_2_^−^ production at 24 h after TBI. Data are expressed as the mean ± SEM. *n* = 6 in each group. ^#^*P* < 0.05 vs. Sham group; ^∗^*P* < 0.05 vs. Tanshinone IIA group. TBI: traumatic brain injury.

NADPH oxidase activation is one of the main mechanisms underlying brain injury. The temporal pattern of NADPH oxidase activation and H_2_O_2_ generation was examined in the adult rat cerebral tissue samples following TBI by controlled cortical contusion. As shown in Figures 7(a) and 7(b), mild TBI increased NADPH oxidase activity and H_2_O_2_ levels at 24 h, but the trend increased at 48 h-72 h after TBI.

Subsequently, it was investigated whether the increase in H_2_O_2_ generation in neurons following TBI was due to activation of NADPH oxidase. This phenomenon was examined by investigating the effect of a NADPH inhibitor, apocynin (4 mg/kg intraperitoneally administered20 min prior to TBI), on H_2_O_2_ generation in brain tissue following TBI. As shown in Figures 7(c)–7(g), pretreatment with apocynin markedly attenuated the generation of H_2_O_2_ at 24 h after TBI compared with the Sham group, suggesting that NADPH oxidase served a critical role in H_2_O_2_ production in neurons following TBI. The present findings suggested that NADPH oxidase was involved in the generation of H_2_O_2_ following TBI.

### 3.6. Effects of Tanshinone IIA on the Expression Levels of p47phox, gp91phox, and Rac1 following TBI

**Figure 8:**
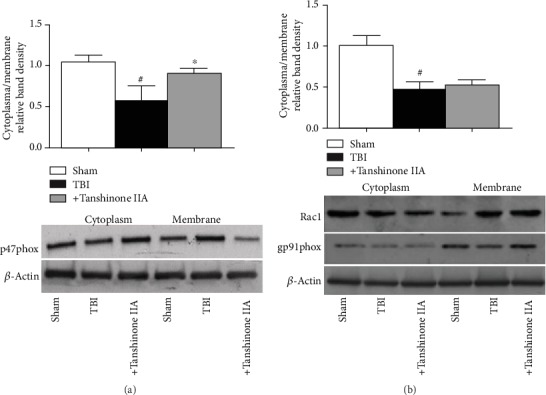
Effects of Tanshinone IIA on the expression levels of p47phox, gp91phox, and Rac1 following TBI. (a) Western blotting analysis of p47phox translocation from cytoplasm to membrane. (b) Western blotting analysis of gp91phox and Rac1expressions. *β*-Actin was used as the loading control. Bands were analyzed by densitometric analysis. Data are expressed as the mean ± SEM. *n* = 6 in each group. ^#^*P* < 0.05 vs. Sham group; ^∗^*P* < 0.05 vs. Tanshinone IIA group. TBI: traumatic brain injury.

A previous study reported that p47phox is the main subunit of NADPH oxidase, which is mainly expressed in the nervous system, particularly in the microglia and the spinal cord, and is involved in ROS formation. As shown in Figures 8(a) and 8(b), p47^phox^ subunit activation was reduced by Tanshinone IIA treatment at 24 h after TBI. However, Tanshinone IIA did not affect the translocation of Rac1 and the level of gp91phox expression. The present results suggested that Tanshinone IIA decreased NADPH oxidase activation via the inhibition of p47phox translocation.

## 4. Discussion

**Figure 9:**
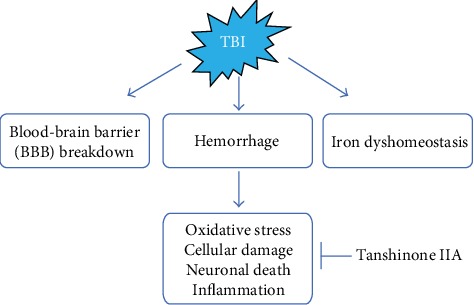
The consequence of Tanshinone IIA on TBI.

The present study investigated the effect of Tanshinone IIA on TBI and its underlying molecular mechanism. The present results suggested that Tanshinone IIA treatment significantly attenuated edema formation and decreased vascular permeability, inhibited inflammation, and reduced apoptosis, thus alleviating TBI-induced damage (Figure 9). Importantly, Tanshinone IIA effects were found to be associated with the inhibition of NADPH oxidase. The present results provided insight into the mechanisms underlying Tanshinone IIA function and indicated that Tanshinone may be a novel treatment to attenuate TBI.

TBI is a leading cause of mortality and disability worldwide. The outcomes of TBI are often related to excitotoxicity, inflammation, metabolic dysfunction, oxidative stress, cellular necrosis, and apoptosis [21, 22]. In addition, its mechanisms are associated with the release of reactive oxygen species (ROS), edema formation, BBB breakdown, release of excitatory amino acids, and acute inflammatory response [23]. Edema formation and brain swelling are considered the most important symptoms of TBI. Brain tissue edema contributes to increase brain volume and intracranial pressure, impairing cerebral circulation and oxygenation, thus worsening ischemic injuries. AQP4 is the main water transporter in the brain and is involved in edema formation in TBI [24]. Previous studies showed that AQP4 regulates water homeostasis in BBB and is involved in the formation of the vasogenic edema, astrocyte migration, and neuronal excitability associated with TBI [25, 26]. Recent studies demonstrated that AQP4 deletion reduces brain edema formation after ischemia stroke in mice. AQP4 knockdown showed improved survival time compared with wide-type mice in a brain edema model. The use of AQP4-null mice provided strong evidence for AQP4 involvement in cerebral water balance. AQP4-null mice are protected from cellular (cytotoxic) brain edema produced by water intoxication, brain ischemia, or meningitis [26]. However, AQP4 deletion aggravates vasogenic (fluid leak) brain edema formed following intraparenchymal fluid infusion or brain abscess [25]. These previous studies suggested that the expression levels of AQP4 are associated with the integrity of the BBB. In the present study, Tanshinone IIA was found to exhibit protective effects on the integrity of BBB structure and function in an animal model of TBI. The present study found that AQP4 expression was significantly upregulated following TBI, and it was associated with an accumulation of Evans blue in the brain and with impaired BBB function. The TBI-induced upregulation of AQP4 was significantly attenuated in the presence of Tanshinone IIA. Moreover, previous studies demonstrated that TBI activates reactive astrogliosis, which is characterized by rapid synthesis of GFAP [27]. Additional studies demonstrated that GFAP can be used as a prognostic tool following severe TBI. Based on these previous studies, GFAP was hypothesized to be involved in the prediction of TBI severity. In the present study, TBI significantly upregulated GFAP. Moreover, the upregulation of GFAP was decreased by Tanshinone IIA treatment. The present results suggested the protective effects of Tanshinone IIA on promoting the recovery of impaired BBB by decreasing the protein expression levels of factors involved in BBB breakdown following TBI.

Microglia are the most important innate immune cells in the CNS and are involved in the process of inflammation serving an important role in nervous system diseases [28, 29]. Microglia respond rapidly to changes in CNS microenvironment and pathological events, and these immune cells were shown to exhibit deleterious and beneficial roles in neuronal damage, phagocytizing necrotic cells and tissue fragments, and activating or inhibiting numerous inflammatory mediators to maintain homeostasis [30]. Previous studies showed that activated microglia express high levels of CD11b, inducing the secretion of proinflammatory factors such as IL-1*β* and TNF-*α* [31]. Among the other proinflammatory factors released by activated microglia, inducible nitric oxide synthase, IL-6, and CCL2 aggravate brain damage. Microglia activation is considered as the initiator of the inflammatory response following brain damage. Several lines of studies showed that microglia activation is involved in the mediation of inflammation following TBI, contributing to neuronal damage via the release of IL-1*β*, TNF-*α*, and IL-6. Previous studies showed that activated microglia exerted neurotoxicity under certain conditions such as ischemia. These previous studies demonstrated that microglia activation increases the releases of IL-1*β*, TNF-*α*, and IL-6, further stimulating the production of matrix metalloproteinases and influencing the permeability of the BBB, inducing secondary brain edema. Therefore, these cytokines are considered to accelerate brain tissue damage following microglia activation. Inhibition of microglia activation was shown to prevent the release of inflammatory cytokines. The present study suggested that TBI induced CD11 upregulation and increased the release of IL-1*β*, TNF-*α*, and IL-6, which are neurotoxic. The present study suggested that microglia activation is significantly reduced in neurons following TBI and Tanshinone IIA treatment. These hints suggested that Tanshinone IIA could directly prevent microglia activation by decreasing apoptosis.

Accumulating evidence demonstrated that oxidative stress is considered the key contributor to secondary injury in the pathophysiology of TBI and oxidative stress is involved in the development of cerebral edema, inflammation, and secondary neuronal damage [32–34]. ROS are considered double-edged swords, since they can maintain cellular homeostasis in physiological conditions and aggravate certain pathological conditions, such as brain injury. NADPH oxidase is the main source of ROS in the brain tissue, and the activation of NADPH oxidase can deteriorate the state of TBI [35]. Overactivation of NADPH oxidase is an important mechanism underlying the increase in oxidative stress, which is associated with the occurrence and development of various diseases. NADPH oxidase is a multicomponent enzyme comprising membrane-bound cytochrome b558 (p22phox and gp91phox heterodimer) and cytosolic regulatory proteins (p67phox, p47phox, p40phox, and Rac2 GTPase). Under pathophysiological conditions, the activation of NADPH oxidase mainly relies on the phox subunits such as p47phox, which translocate to the membrane. Accumulating evidence demonstrated that the NADPH oxidase activation contributes to aggravate brain injury by mediating oxidative stress and microglia activation following TBI [36]. Additional studies showed that the inhibition of NADPH oxidase could decrease excessive ROS production following TBI, preventing programmed neuronal death and microglia activation [37]. Present results suggested that the expression level of p47phox was upregulated following TBI and the administration of Tanshinone IIA inhibited p47phox translocation. These results suggested that Tanshinone IIA reduced the TBI-associated overproduction of ROS through the inhibition of p47phox translocation.

Numerous studies demonstrated that ROS accumulation induced by TBI is a critical factor in brain damage [38]. Moreover, ROS overproduction is involved in the initiation of programmed cell death following TBI. Importantly, neuronal apoptosis may be a result of oxidative stress [37–39]. Considering the important role of oxidative stress in TBI, the investigation of ROS antioxidants or scavengers may provide novel strategies to inhibit TBI-induced cell apoptosis [32]. Tanshinone IIA is a potent antioxidant, and it is useful in the treatment of cardiovascular and cerebrovascular diseases. In particular, Tanshinone IIA can scavenge lipid free radicals, thus decreasing cytotoxicity *in vitro* and *in vivo*. In the present study, Tanshinone IIA showed antioxidative effects following TBI, including the increase in SOD, CAT, and GSH-PX activities, and decrease in MDA content, in line with previous studies. Furthermore, Tanshinone IIA treatment inhibited TBI-induced neuronal apoptosis *in vivo*. The present results suggested that the protective effects of Tanshinone IIA against TBI may involve its potent antioxidative activity.

Collectively, the present results suggested that Tanshinone IIA had neuroprotective properties including improved brain tissue edema formation, decreased the release of inflammatory mediators, and reduced oxidative damage and apoptosis via the inhibition of NAPDH oxidase activation. The present results suggested that Tanshinone IIA may facilitate the development of novel therapeutic and preventive strategies for the treatment of TBI.

## Data Availability

The data used to support the findings of this study are available from the corresponding author upon request.
